# Polydextrose: Physiological Function, and Effects on Health

**DOI:** 10.3390/nu8090553

**Published:** 2016-09-08

**Authors:** Mariane Moreira Ramiro do Carmo, Julia Clara Leite Walker, Daiana Novello, Valeria Maria Caselato, Valdemiro Carlos Sgarbieri, Arthur C. Ouwehand, Nelson Adami Andreollo, Priscila Aiko Hiane, Elisvânia Freitas dos Santos

**Affiliations:** 1 Food Technology and Public Health Division, Center of Biological Sciences and Health, Federal University of Mato Grosso do Sul, Campo Grande 79070-900, Mato Grosso do Sul, Brazil; mariane-nutricionista@hotmail.com (M.M.R.d.C.); juliaclaraw@gmail.com (J.C.L.W.); priscila.hiane@ufms.br (P.A.H.); 2 Sector of Health Sciences, Department of Nutrition, State University of Centro-Oeste, Guarapuava 85040-080, Paraná, Brazil; nutridai@gmail.com; 3 Institute of Nutrition Josué de Castro, Federal University of Rio de Janeiro, Rio de Janeiro 21941-901, Rio de Janeiro, Brazil; valcaselato@gmail.com; 4 School Food Engineering, State University of Campinas, Campinas 13083-862, São Paulo, Brazil; sgarb@fea.unicamp.br; 5 Active Nutrition, DuPont Nutrition & Health, Kantvik 02460, Finland; arthur.ouwehand@dupont.com; 6 School of Medical Sciences, State University of Campinas, Campinas 13083-887, São Paulo, Brazil; nandreollo@hotmail.com

**Keywords:** polydextrose, prebiotics, health, functional foods, fiber

## Abstract

Polydextrose (PDX) is a non-digestible oligosaccharide used widely across most sectors of the food industry. It is a randomly linked glucose oligomer containing small amounts of sorbitol and citric acid. The random bonds in PDX prevent mammalian digestive enzymes from readily hydrolyzing the molecule and it has a reported energy value of 1 kcal/g. These properties have led to the acceptance in many countries that PDX provides similar physiological effects as other dietary fibers and has shown prebiotic potential. Dietary intervention with prebiotics has been shown to selectively stimulate the growth and/or activity of one or a limited number of intestinal bacteria associated with several physiological benefits on health. Therefore, the objective of this review was a survey of the literature on the effect of supplementation with PDX in health, and to list the benefits for maintaining health and/or reducing the development of diseases.

## 1. Introduction

In recent years, scientists have become aware that the human microbiota, in general, and the gut microbiota, in particular, play a major role in health and diseases, such as obesity and diabetes, among others. A large amount of evidence has come to light regarding the beneficial effects, either for the host or the gut microbiota, of some foods and food ingredients or biochemical compounds. Probiotics and prebiotics designed to manipulate the gut microbiota for improving health outcomes are in demand as the importance of the gut microbiota in human health becomes increasingly established [1,2]. Probiotics are live microorganisms that, when administered in adequate amounts, confer a health benefit on the host [3]. Prebiotics, on the other hand, may be normal constituents of the diet or added to functional foods [4].

The concept of prebiotics was first introduced by Gibson and Roberfroid [5] as “non-digestible food ingredients that beneficially affect the host by selectively stimulating the growth and/or activity of one or a limited number of bacteria in the colon, and thus improves host health”. Prebiotics are sometimes referred to as non-digestible oligosaccharides (NDOs) and fiber [5,6]. However, it should be noted that not all dietary fibers are prebiotics as they may not be (selectively) fermented by members of the intestinal microbiota. Roberfroid et al. [7] updated these ideas as follows: “the selective stimulation of growth and/or activity(ies) of one or a limited number of microbial genus(era)/species in the microbiota that confer(s) health benefits to the host”. For a dietary compound to be classified as a prebiotic, at least three criteria need to be fulfilled: (1) the component must not be hydrolyzed or absorbed in the stomach or small intestine; (2) it must be a selective substrate for beneficial commensal bacteria in the colon, such as bifidobacteria; and (3) fermentation of the component should induce beneficial luminal/systemic effects within the host [8].

Responses to the fermentation of prebiotics are affected by individual variation in the microbiota as well as by the chemical structure of the specific carbohydrates [6]. Both gas production and prebiotic properties of the carbohydrates are likely to be influenced by the monosaccharide composition (commercially used prebiotics are primarily composed of glucose, fructose, galactose, or xylose), glycosidic linkages between the monosaccharide residues, and the degree of polymerization (DP) of the prebiotic. Several studies have suggested that more complex substances with longer chain lengths (DP > 10) and diverse linkages are fermented more slowly, with less gas production, and with potentially less bifidogenic capacity than short-chain carbohydrates (DP < 10) [9,10]. In vitro gas and short-chain fatty acid (SCFA) production data show that short-chain oligosaccharides are more rapidly fermented and produce more SCFA and gas than substrates with greater DP [9,10]. Inulin, that typically has a DP between 3 and 60, is an exception and has a prebiotic potential due to its high SCFA production and bifidogenicity, but also causes gas production [11,12,13].

Among the non-digestible food ingredients studied for their prebiotic potential, fructo-oligosaccharides (FOS; oligofructose), inulin, and galacto-oligosaccharides (GOS) are widely accepted as prebiotics, a fact supported by many human trials [7]. Studies suggest that also polydextrose (PDX) has prebiotic potential [14,15]. Therefore, the objective of this review was to survey the literature on the effect of dietary supplementation with PDX on health, and to list the benefits in preventing and/or reducing the risk of the development of diseases.

## 2. Polydextrose 

PDX is a highly branched, randomly bonded glucose polymer with an average DP of 12, ranging from 2 to 120. The molecule contains all possible combinations of α- and β-linked 1→2, 1→3, 1→4, and 1→6 glycosidic linkages, though the 1→6 (both α and β) predominates [16,17,18]. Due to its complex structure, PDX is not hydrolyzed by mammalian digestive enzymes in the small intestine, passing intact into the colon, in which it is gradually and partly fermented by the endogenous microbiota and the remainder, approximately 60%, is excreted in the feces [19,20,21]. Since PDX is not utilized by the host, energy is only provided by the SCFA produced from its partial fermentation by the microbiota. This results in an energy contribution of 1 kcal/g [20,22,23,24].

PDX is not sweet, has a neutral taste, and can be used as a low-calorie bulking agent in a wide range of foods, such as baked goods, confectionery, dairy products, and functional beverages as it is highly soluble in water and results in a non-viscous solution. PDX has been the subject of many studies, due to its versatility and multifunctionality. Besides being an excellent ingredient, it has been approved for use in foods in over 60 countries and is recognized as a dietary fiber in more than 20 countries [15,17,25,26]. It has been shown that daily intake of 4–12 g PDX improves physiologic functions without adverse effects [14].

## 3. Effects on Mineral Absorption

Iron is normally absorbed in the small intestine with the stomach playing an essential role in improving the biological availability of iron [27,28,29]. Also, calcium is normally absorbed in the small intestine [30]. However, when its absorption pathway is not sufficient to cover the body’s needs, these minerals can also be absorbed from the colon. As will be discussed below (Section 4), PDX has been documented to be slowly and gradually fermented, and the beneficial effects of PDX are mediated either through the metabolites produced and/or altered microbiota composition [31]. The production of SCFA will lead to a lowering of the luminal pH in the colon and make calcium more soluble. For iron, some chelation may happen with the SCFA, which may also facilitate passive absorption [31,32].

Legette et al. [33] demonstrated in a postmenopausal rodent model that the increased production of SCFAs after consumption of PDX increased mineral absorption in the colon. The animals received 5 g/100 g PDX for 4 weeks, and subsequently, a significant increase in the acute retention of calcium (102%) and magnesium (56%) was seen in animals fed diets supplemented with PDX when compared to the control group (Table 1).

In another study conducted by Albarracín et al. [34], the bioavailability of calcium was evaluated in growing male Wistar rats. After 60 days, the animals who had received PDX supplementation showed apparent absorption of calcium (calculated on the basis of food intake and fecal excretion) that was significantly higher (16%) when compared to the control group, along with increased bone mineral density in the femur (7%), tibia (9%), and spine (7%) (Table 1). These data corroborated with the results obtained by Weaver [35], who observed an increase in bone calcium absorption (262%) in rats after 12 weeks of supplementation with PDX, when compared to the control group (Table 1).

Santos et al. [30] and Santos et al. [27] evaluated the effects of 8 weeks administration of PDX on absorption of calcium and iron in partially gastrectomized rats. The animals were fed diets with PDX or without PDX (controls). Rats fed a diet containing PDX showed an increase in calcium (22%) [30] and apparent absorption of iron (74%) [27], followed by a higher serum iron concentration (52%), hematocrit (34%), and hemoglobin (16%) (Table 1). PDX also increased the concentration of calcium in the bones (16%) of totally gastrectomized rats [36], and thus, PDX can help with the absorption of calcium by the host and possibly reduce the risk of osteoporosis (Table 1). It seems plausible that a common mechanism via the paracellular pathway is involved in saccharide-induced calcium absorption in the epithelium of the gastrointestinal tract [37]. Therefore, it is possible to identify components and/or functional food ingredients, such as PDX, that can influence the absorption of calcium and iron, ensuring better bioavailability.

## 4. Effects on Microbiota

In vitro studies have indicated that PDX has prebiotic potential [15,19,20,32,38]. PDX has been shown to beneficially modify the colonic microbial composition and activity. In contrast to other prebiotics with smaller molecular weights, PDX is slowly fermented and remains available as a carbon source for the microbiota throughout the colon, including the distal part of the colon. The sustained and slow fermentation of PDX has been demonstrated in vitro, in vivo, and in human dietary intervention trials [31]. The gradual fermentation by the colonic microbes leads to a sustained production of SCFA (acetate, propionate, and butyrate) and minor amounts of gas [7,8,9,10,15,17,38]. The increased amounts of SCFA in the more distal part of the colon may mediate the beneficial effects connected with PDX consumption, absorption of minerals from the colon, and improved gastrointestinal function, e.g., relief of constipation and softer stools in humans (more about this is subsequent sections) [20,22,31,32,39,40]. The slow and sustained fermentation most likely explains the good tolerance of PDX observed in human intervention studies [39]. It also ensures that PDX is present in the distal part of the colon, where it decreases proteolytic fermentation that would otherwise take place once substrates for saccharolytic fermentation are depleted [16,41].

In a recent randomized, double-blind, placebo-controlled study with healthy adult males, Holscher et al. [19] evaluated the impact of supplementation of 21 g/day of PDX on fecal metabolites, bacterial taxa, and bacterial metagenomes of humans. The associations between fecal microbiota and metabolism pathways were identified by 454 pyrosequencing, showing that consumption of PDX significantly suppressed the numbers of the phylum Firmicutes (12%) and significantly increased the numbers of the phylum Bacteroidetes (12%) compared with placebo group (Table 2). These data supported the results obtained by Hooda et al. [20], who conducted a study with a similar experimental setup in healthy men and found that PDX supplementation (21 g/day) during 21 days produced changes in the fecal microbiota of these individuals. The consumption of PDX led to greater fecal numbers of Clostridiaceae (5%) and Veillonellaceae (2%) and lower numbers of Eubacteriaceae (4%) compared with the placebo. The abundance of *Faecalibacterium* (5%), *Phascolarctobacterium* (1%), and *Dialister* (1%) was greater in response to PDX. Hooda et al. [20] concluded that the supplementation of PDX clearly had a positive impact on the bacterial composition of the human intestinal microbiota (Table 2).

Costabile et al. [38] carried out a double-blind crossover, placebo-controlled feeding study in healthy human subjects; the main objective of the study was to identify the microbial groups affected by the fermentation of PDX (8 g/day) in the colon. After 3 weeks, a significant increase in the number of *Ruminococcus intestinalis* (8 log_10_), known as the principal producer of butyrate; decrease in fecal *Lactobacillus*–*Enterococcus* (9 log_10_); and significant reduction of genotoxicity of fecal water by slow fermentation of PDX in the colon were reported (Table 2). Similar results were reported by Forssten et al. [42], when two concentrations of PDX (2% and 4% *v*/*v*) were tested. For 48 h, the test substrates were added to simulated ileal medium before inoculation, and fed in 3 h cycles to a simulated colon model containing the microbiota of healthy volunteers and spiked with viable *Clostridium difficile*. Supplementation with PDX resulted in several changes in groups of colonic microorganisms and a tendency toward decreased strains of *C. difficile* with both concentrations with more than one log (from 8–9 log_10_ to 4–7 log_10_/mL simulation media), supporting the notion that PDX may be able to modulate the composition and/or function of the human colonic microbiota (Table 2). Lamichhane et al. [43] found that dietary supplementation with PDX (8 g/day) for 3 weeks followed by a 3 weeks washout period had a strong differentiating effect on the metabolome of human feces compared to feces from the control period. The high-resolution nuclear magnetic resonance (NMR) of fecal samples revealed a wide range of metabolites in the group supplemented with PDX. A pronounced effect of PDX intake was observed on the NMR profile. Visual inspection of spectra showed that samples collected after PDX intervention had a markedly different fecal metabolite profile than baseline, placebo, and washout samples. The difference can be mainly attributed to the presence of oligosaccharides formed from the partial degradation of PDX. This was particularly positively correlated with fecal *Bifidobacterium* levels (10 log_10_) when compared to placebo (Table 2) [43].

## 5. Effects on Intestinal Cells and Immune Effects

Some fibers may also play a role in improving immune function via production of SCFAs. Prebiotics may be potential chemopreventive agents on the basis of the observation that health-promoting bacteria such as bifidobacteria, that do not produce carcinogenic or genotoxic compounds, but instead produce potentially protective metabolites [44]. Witaicenis et al. [45] evaluated the effects of PDX in a model of rat colitis using 2,4,6-trinitrobenzenesulfonic acid(TNBS) acid-induced intestinal inflammation and its effects on the intestinal anti-inflammatory activity of sulfasalazine. Three groups received 5% PDX dissolved in drinking water for 21 days prior to colitis induction and 4 days thereafter. The results obtained in the present study showed a protective and preventative effect of PDX on the TNBS-induced inflammatory process in rats. Recovery from the TNBS-induced colonic damage in rats treated with PDX was evidenced by a reduction in colonic damage (57%), a counteraction of glutathione (GSH) depletion (35%), and a reduction in myeloperoxidase (MPO) activity (58%) (Table 3). PDX was showed to be an important dietary supplement in the prevention and protection of inflammatory bowel disease, which was associated with improved oxidative stress in intestinal epithelial cells.

Peuranen et al. [46] evaluated the effects of PDX and the sugar alcohol lactitol on gut microbiota, microbial metabolism, and gut immune responses in rats for 3 weeks. PDX increased the secretion of IgA in the cecum (345%). Secretion of IgA increased even more—almost 10-fold—with the combination of PDX + lactitol (996%) when compared with the control group, an important indicator of a potentially improved immune defense [46] (Table 3). Yamamoto et al. [47] evaluated the influence of the intake of non-digestible carbohydrates; FOS or PDX + lactitol during 21 days, on IgA response and the expression of polymeric Ig receptor in the submandibular gland and cecal digest of rats. These authors concluded that non-digestible carbohydrates, such as PDX in combination with lactitol, have an important role in increasing concentrations of IgA in the submandibular gland tissue (25%), saliva (1000%) and cecal digest (212%) (Table 3).

Putaala et al. [48] evaluated in an in vitro assay the protective effect of PDX fermentation against the risk of developing colon cancer. The metabolites after fermentation were applied to colon cancer cells and changes in gene expression were studied. Two concentrations of PDX, 1% and 2% (*v*/*v*), were selected for the analysis as these amounts have a dose-dependent effect in total concentration of SCFAs when compared with the baseline fermentation. The number of genes differentially regulated by 1% PDX metabolome doubled in the 2% exposure with 307 and 710 genes differentially regulated, respectively, which points to a dose-response. The authors observed a differential regulation of key genes in the etiology of cancer by PDX fermentation metabolites, affecting the number of metabolically active cells; moreover, the fermentation of PDX played an important role in inducing apoptosis of colon cancer cells (Table 3). Similar results were reported by Fava et al. [49], who found that the addition of 30 g/day of PDX for 21 days to pigs’ diet significantly changed the composition of fermentation products, especially in the distal colon and reduced the expression of cyclooxygenase (COX)-2 of the mucosa (63%), thereby possibly reducing the risk of developing conditions that promote colon cancer (Table 3).

## 6. Effects on Blood Glucose and Lipid Metabolism

SCFAs, the end-products of fermentation of dietary fibers by the anaerobic intestinal microbiota, have been shown to exert multiple beneficial effects on mammalian energy metabolism [22]. The relatively high number of acute human intervention studies indicating the beneficial effects of PDX on appetite, satiety, and energy intake have attempted to draw the conclusion that PDX is a potent approach for the prevention and treatment of obesity and comorbidities, including reduced risk of cardiovascular disease (CVD), diabetes, hypertension, and gastrointestinal disorders [32,50].

Insulin is an important regulator of lipid metabolism, and therefore, insulin resistance in the peripheral tissues is a link between metabolic syndrome and dyslipidemia [51].

In a recent study, Tiihonen et al. [23] evaluated the effect of PDX supplementation on postprandial triglycerides in normolipidemic, hyperlipidemic, and obese subjects. On two separate occasions all subjects ate two high-fat meals (4293 kJ, 36% from fat), one with PDX (either 12.5 g or 15 g/day) and one without PDX. Each study consisted of two periods (postprandial interventions) and a washout period of approximately 10 days. The maximum postprandial triglyceride concentration was 2 mmol/L in normolipidemic subjects and significantly higher (2 mmol/L) in obese and in hyperlipidemic subjects (3 mmol/L). The responses were significantly (*p* < 0.05) reduced in all three groups when PDX was included in the high-fat meal (Table 4). Albarracin et al. [34] evaluated the effect of diets with different types of fibers on metabolic parameters in growing rats and found that PDX (5 g/100 g diet) considerably decreased the content of triglyceride (17%) and malondialdehyde (MDA; 36%) in liver after 60 days of supplementation (Table 4).

Stenman et al. [52] investigated whether supplementation with probiotics and prebiotics combined with drug therapy for diabetes could affect glucose metabolism in a diabetic mouse model. Glucose metabolism was assessed at 4 weeks, and it was observed that compared to sitagliptin (SITA) only, PDX supplementation (0.25 g/day) combined with sitagliptin (PDX + SITA) or with probiotic *Bifidobacterium animalis* subsp. *lactis* 420 (B420) and SITA (PDX + B420 + SITA) significantly decreased fasting plasma glucose levels (40% and 49%, respectively). Furthermore, glucose response in the oral glucose tolerance test was reduced 28% more with SITA + PDX compared to SITA only. Thus, the combination of probiotics and/or prebiotics with an antidiabetic drug improves glycemic control and insulin sensitivity in mice (Table 4). Konings et al. [53] evaluated the effect of substituting 30% of available carbohydrates with PDX at breakfast and lunch in overweight men and women who underwent four different dietary interventions, with a 1 week washout period between the interventions. They found that this led to reduced glucose peaks, which was accompanied by reduced postprandial insulin responses. The peak glucose response after breakfast was lower upon consumption of the PDX diet than after consumption of the full energetic (FULL) diet (FULL vs. PDX, *p* = 0.06). After breakfast and lunch, the insulin response was significantly lower after consumption of the PDX than after consumption of the FULL diet (FULL vs. PDX, *p* = 0.02). Moreover, the authors observed higher circulating concentrations of non-esterified fatty acids simultaneously with increase in fat oxidation over 24 h, which could be attributed to the low caloric value of PDX. PDX also exerted a suppressive effect in appetite control (Table 4). Higher circulating fatty acid concentrations, subsequent increased fat oxidation, and in turn, increased postprandial fat oxidation rate may impact fat storage and satiety and may thereby beneficially affect weight control in the long term [54]. Furthermore, it may result in decreased ectopic fat accumulation, which is associated with improved insulin sensitivity and glucose metabolism [55].

## 7. Effects on Bowel Function

One of the main physiological effects of dietary fiber that is noted by the consumer is its stimulation of bowel function. Shimada et al. [56], in a randomized, placebo-controlled study, evaluated the bowel habits of Japanese hemodialysis patients, induced by the ingestion of 10 g/day PDX for 8 weeks. The authors reported that PDX reduced bowel transit time (90%) and increased total weekly bowel frequency (from 3 times to 7 times a week) without inducing adverse gastrointestinal symptoms such as abdominal pain or bloating or induction of diarrhea (Table 5). Furthermore, PDX improved the ease of bowel movements by producing soft stools. Hengst et al. [57] demonstrated in healthy adults that a daily intake of 8 g PDX during 2 weeks shortened transit time oro–fecal (22%) in subjects suffering from constipation (Table 5). Also, Costabile et al. [38] observed that consumption of 8 g/day PDX during 3 weeks induced changes in bowel habits of healthy individuals, such as less abdominal discomfort (22%) and a tendency toward the formation of softer stools (19%) when comparing PDX and placebo groups.

Magro et al. [58] evaluated the synergistic effect of yogurt supplemented with PDX, *Lactobacillus acidophilus*, and *Bifidobacterium lactis* HN019 on intestinal transit in constipated subjects over a period of 14 days, and observed that individuals receiving supplemented yogurt had a significantly reduced transit time in the colon (35%) compared to the control group (Table 5). Results supporting these data were earlier reported by Ribeiro et al. [59], who evaluated the effect of a prebiotic blend of PDX and GOS, 0.5 g of each per serving (PDX/GOS) in an infant formula with cow’s milk on the gastrointestinal tract of healthy children aged 9–48 months. The latter authors demonstrated that the addition of PDX + GOS in formula for 108 days was well tolerated and induced a pattern of more frequent (172%) and softer stools than control group, and that this may therefore have an important application in the management and prevention of functional constipation (Table 5).

Although PDX has been observed to affect bowel function, the risk of excessive bowel movements is small. The Joint FAO/WHO Expert Committee on Food Additives (JECFA) and the European Commission Scientific Committee for Food (EC/SCF) concluded that PDX has a mean laxative threshold of ~90 g/day (1.3 g/kg per week) or 50 g as a single dose [25]. Thus, the margins between a beneficial effect and overdosing are wide and indicate good tolerance.

## 8. Effects on Energy Intake

Fibers have a tendency to influence satiation (the feeling of fullness that leads to termination of a meal) and satiety (the time after a meal until one is hungry again) [60]. A recent meta-analysis, Ibarra et al. [61] showed that desire to eat during the satiation period favors PDX for the reduction of this subjective feeling of appetite (SMD = 0.24); this effect was also significant when performing the subanalysis, based on sex, for the male population (SMD = 0.35). This correlates very well with the results of an earlier meta-analysis on the influence of PDX on energy intake [62]. Included in a midmorning snack, PDX leads to a significantly reduced energy intake at the subsequent lunch. This effect was observed to be dose-dependent; 5% less energy intake with a preload of 6.25 g PDX and up to 17% less energy intake with 25 g of PDX. Importantly, this did not lead to a compensation of energy intake during the following dinner. Thus the overall energy intake for the whole day was reduced, also in a dose-dependent manner [62].

## 9. Final Considerations

The key characteristics of dietary fiber are effects on nutrient absorption, changes in intestinal microbiota composition and/or activity, modulation of immune function, improving postprandial serum glucose and lipid responses, improving bowel function, and influencing energy intake. Many of these physiological benefits are interrelated and SCFA play a central role here. In the present review, we present data that indicate that PDX shows evidence for several of these areas; in particular for the postprandial benefits, energy intake, and bowel function. For other areas, such as nutrient absorption and immune modulation, emerging evidence exists, but more studies, especially human studies, are required. Finally, although the microbiota, through production of SCFA plays an important role in the observed benefits of PDX, at the present stage of science it is difficult to indicate what microbial groups should be stimulated; suppression of certain known pathogens is more clear, but this will have little relevance for the general healthy population.

## Figures and Tables

**Table 1:** The effects of administration of polydextrose on mineral absorption.

Host	Dose	Effects	Reference
Effects on mineral absorption			
Rats	5 g/100 g	- Increased in the acute retention of calcium (102%). - Increased the acute retention of magnesium (56%).	Legette et al. [33]
Rats	5 g/100 g	- Increased in apparent calcium absorption (16%). - Increased in bone mineral density (femur (7%), tibia (9%) and spine (7%).	Albarracín et al. [34]
Rats	4 g/100 g	- Increased in bone absorption of calcium (262%).	Weaver [35]
Rats	5 g/100 g	- Increased in bone absorption of calcium (22%).	Santos et al. [30]
Rats	5 g/100 g	- Increased in bone absorption of calcium (16%).	Hara et al. [36]
Rats	5 g/100 g	- Increase in apparent iron absorption (74%). - Increased serum iron concentration (52%), hematocrit (34%) and hemoglobin (16%).	Santos et al. [27]

Effects of administration of different doses of polydextrose (PDX) in different experimental models on mineral absorption.

**Table 2:** The effects of administration of polydextrose on microbiota.

Host	Dose	Effects	Reference
Effects on microbiota			
Humans	21 g/day	- Suppressed the numbers of the phylum Firmicutes (12%) compared with placebo group. - Increased the numbers of the phylum Bacteroidetes (12%) compared with placebo group.	Holscher et al. [19]
Humans	21 g/day	- Increased fecal Clostridiaceae (5%), Veillonellaceae (2%), *Faecalibacterium* (5%), *Phascolarctobacterium* (1%), and *Dialister* (1%) compared with a placebo. - Decrease *Eubacteriaceae* (4%) compared with a placebo.	Hooda et al. [20]
Humans	8 g/day	- Increase in *Ruminococcus intestinalis* (8 log_10_). - Decrease in *Lactobacillus-Enterococcus* (9 log_10_).	Costabile et al. [38]
In vitro	2% or 4% (*v*/*v*)	- Reduced development of *Clostridium difficile* (from 8–9 log_10_ to 4–7 log_10_/mL). - Modulation of colonic microbiota composition.	Forssten et al. [42]
Humans	8 g/day	- Increase in *Bifidobacterium* (10 log_10_).	Lamichhane et al. [43]

Effects of administration of different doses of polydextrose (PDX) in different experimental models on microbiota.

**Table 3:** The effects of administration of polydextrose on intestinal cells and immune effects.

Host	Dose	Effects	Reference
Effects on intestinal cells and immune effects			
Rats	5 g/100 mL	- Reduction in colonic damage (57%). - Depletion a counteraction of glutathione (GSH) (35%). - Reduction in myeloperoxidase (MPO) activity (58%).	Witaicenis et al. [45]
Rats	2 g/100 g	- Increase secretion of IgA (345%).	Peuranen et al. [46]
Rats	2.5 g/100 g PDX + 2.5 g/100 g lactitol	- Increased in luminal IgA concentration in the submandibular gland tissue (25%), saliva (1000%) and cecal digest (212%).	Yamamoto et al. [47]
In vitro	2% *v*/*v*	- Increase the number of genes differentially regulated by 2% PDX metabolome (131%).	Putaala et al. [48]
Pigs	30 g/day	- Decreased expression of cyclooxygenase (COX)-2 in the distal colon (63%).	Fava et al. [49]

Effects of administration of different doses of polydextrose (PDX) in different experimental models on intestinal cells and immune effects.

**Table 4:** The effects of administration of polydextrose on blood glucose and lipid metabolism.

Host	Dose	Effects	Reference
Effects on blood glucose and lipid metabolism			
Humans	12.5 or 15 g/day	- Significant (*p* < 0.05) decreased postprandial triglyceride response: in normolipidemic, obese and hyperlipidemic subjects with PDX in high fat meal.	Tiihonen et al. [23]
Rats	5 g/100 g diet	- Decreased the content of triglyceride (17%) and malondialdehyde (36%) in liver when compared to the control group.	Albarracín et al. [34]
Mice	PDX + B420 + SITA (0.25 g/day + 109 CFU/day) or PDX + SITA (0.24 g/day + 3 mg/kg (day))	- SITA + PDX reduced glycaemia in the oral glucose tolerance test significantly more than SITA only (28%). - PDX or B420 + PDX, together with SITA, decreased fasting glucose concentrations compared to SITA only (40% and 49%, respectively, for each comparison).	Stenman et al. [52]
Humans	56.7 g/day	- Peak glucose response after breakfast was lower after consumption of PDX diet than after consumption of the FULL diet (FULL vs. PDX *p* = 0.06). - After breakfast and lunch, the insulin response was significantly lower after consumption of the PDX than after consumption of the FULL diet (FULL vs. PDX *p* = 0.02).	Konings et al. [53]

Effects of administration of different doses of polydextrose (PDX) in different experimental models on blood glucose and lipid metabolism. PDX: polydextrose; B420: *Bifidobacterium animalis* subsp. *lactis* 420; SITA: sitagliptin.

**Table 5:** The effects of administration of polydextrose on bowel function.

Host	Dose	Effects	Reference
Effects on bowel function			
Human	10 g/day	- Reduced transit time (90%) in hemodialysis patients. - Increased frequency of bowel movements (150%) in hemodialysis patients.	Shimada et al. [56]
Human	8 g/day	- Shortened oro–fecal transit time (22%) in subjects suffering from constipation.	Hengst et al. [57]
Human	8 g/day	- Reduced abdominal discomfort (22%) and a tendency toward the formation of softer stools (19%) in healthy individuals.	Costabile et al. [38]
Human	3.6 g/day	- Reduced transit time (35%) in subjects suffering from constipation.	Magro et al. [58]
Human (Children)	PDX and GOS, 0.5 g of each per serving (PDX/GOS)	- Induced a pattern of more frequent stools (172%) in healthy children.	Ribeiro et al. [59]

Effects of administration of different doses of polydextrose (PDX) in different experimental models on bowel function.

## Abbreviations

The following abbreviations are used in this manuscript:

PDX	Polydextrose
NDOs	Non-digestible oligosaccharides
DP	Degree of polymerization
SCFA	Short-chain fatty acid
FOS	fructo-oligosaccharides
GOS	Galacto-oligosaccharides
COX-2	Cyclooxygenase
CVD	Cardiovascular disease
SCF	Soluble corn fiber
NMR	High-resolution nuclear magnetic resonance
SITA	Sitagliptin
B420	Bifidobacterium animalis subsp. lactis 420
GSH	Glutathione
TNBS	Trinitrobenzenesulfonic
MPO	Myeloperoxidase
MDA	Malondialdehyde
